# Vibrational Polaritons in Disordered Molecular Ensembles

**DOI:** 10.1021/acs.jpclett.2c02341

**Published:** 2022-08-31

**Authors:** Bar Cohn, Shmuel Sufrin, Arghyadeep Basu, Lev Chuntonov

**Affiliations:** ^†^Schulich Faculty of Chemistry, ^‡^Solid State Institute, ^§^Faculty of Mechanical Engineering, Technion−Israel Institute of Technology, Haifa 3200003, Israel

## Abstract

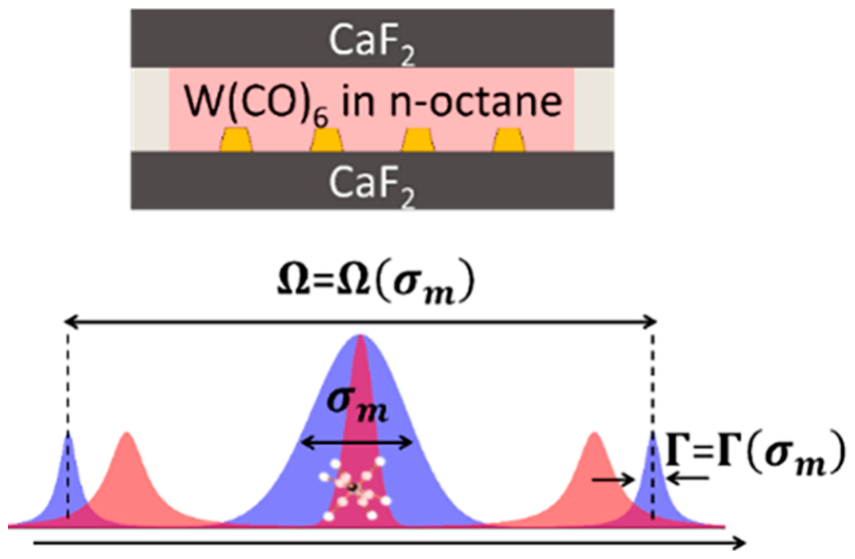

Disorder is an intrinsic attribute of any realistic molecular system. It is known to lead to localization, which hampers efficient transport. It was recently proposed that in molecular ensembles strongly coupled to photonic cavities, moderate disorder leads to delocalization and increases of the transport and chemical reaction rates. Vibrational polaritons involve molecular vibrations hybridized with an infrared cavity. When the coupling strength largely exceeds the molecular inhomogeneity, polaritons are unaffected by disorder. However, in many experiments, such a homogeneous limit does not apply. We investigated vibrational polaritons involving molecular ensembles with systematically modified disorder. Counterintuitively, moderate disorder leads to an increase in Rabi splitting and the modification of the polariton bandwidths. Experimental spectroscopic data agree with a Tavis–Cummings-like model that suggests enhanced delocalization of the reservoir states occurs via the admixture of the cavity mode. Our results provide new insights into the paradigm of disorder-induced cavity-assisted delocalization in molecular polaritons.

Molecular polaritons are hybrid light–matter states formed when an ensemble of *N* molecular transitions strongly interacts with the “privileged” electromagnetic mode of an optical cavity.^1,2^ Polaritons are envisioned to play revolutionary roles in emerging applications ranging from quantum technologies to chemical catalysis;^3−8^ thus, they have been investigated extensively.^9−11^ Experimental results are frequently analyzed within the framework of cavity quantum electrodynamics. Specifically, the Tavis–Cummings (TC) model,^12,13^ which describes the interaction between a cavity and an ensemble of degenerate molecular modes, predicts that a manifold of the reservoir states, potentially with new and important properties, is formed in addition to a pair of polariton states.^14−17^

Recently, the TC model was generalized to account for molecular disorder.^18−23^ Disorder is an intrinsic property of any realistic molecular system, which one expects to affect the composition and dynamics of both polaritons and reservoir states.^18−20,24,25^ When the molecule–cavity coupling strength largely exceeds the molecular inhomogeneous bandwidth, the effect of the disorder can be neglected.^26^ However, when the ratio between the two is smaller than about an order of magnitude, which is a frequent situation in polaritonic systems involving molecular ensembles,^27−29^ the conclusions drawn, based on the application of homogeneous models, might not rigorously hold. Recent studies theorize that reservoir states delocalize over multiple molecules in the presence of disorder, thus facilitating efficient transport^18,25,30^ and vacuum-field catalysis,^20,31^ which is strikingly opposed to the typical effect that disorder has in the absence of coupling to a photonic cavity.^32^ However, in most practical realizations of these systems, experimentally controlling the molecular disorder is formidable, and no direct experimental demonstration of the associated effects has been reported.

Consider the first excitation tier of the system schematically illustrated in Figure 1a, where an ensemble of noninteracting two-level systems  (), representing molecular transitions, is coupled with rates  to a cavity mode . The corresponding TC-like Hamiltonian^18−22,25^ is
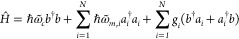
1where  and  are the molecular and photonic creation operators. To account for intrinsic losses,  and  are assumed to be complex, , where  is the dissipation rate associated with the homogeneous bandwidth of the corresponding transition.^33^ The values  are normally distributed about the mean  with the standard deviation (inhomogeneous bandwidth) . For uniform values , a transformation can be made to the so-called bright–dark states basis (Figure 1a),^11,13,18,19^ where the molecular manifold is transformed into a many-body superoscillator bright state  and a manifold of states , where  is the excited state of molecule *j* and  Here,  is the only state that directly couples to the cavity with the collective rate , whereas  are coupled to  with rates .

**Figure 1:**
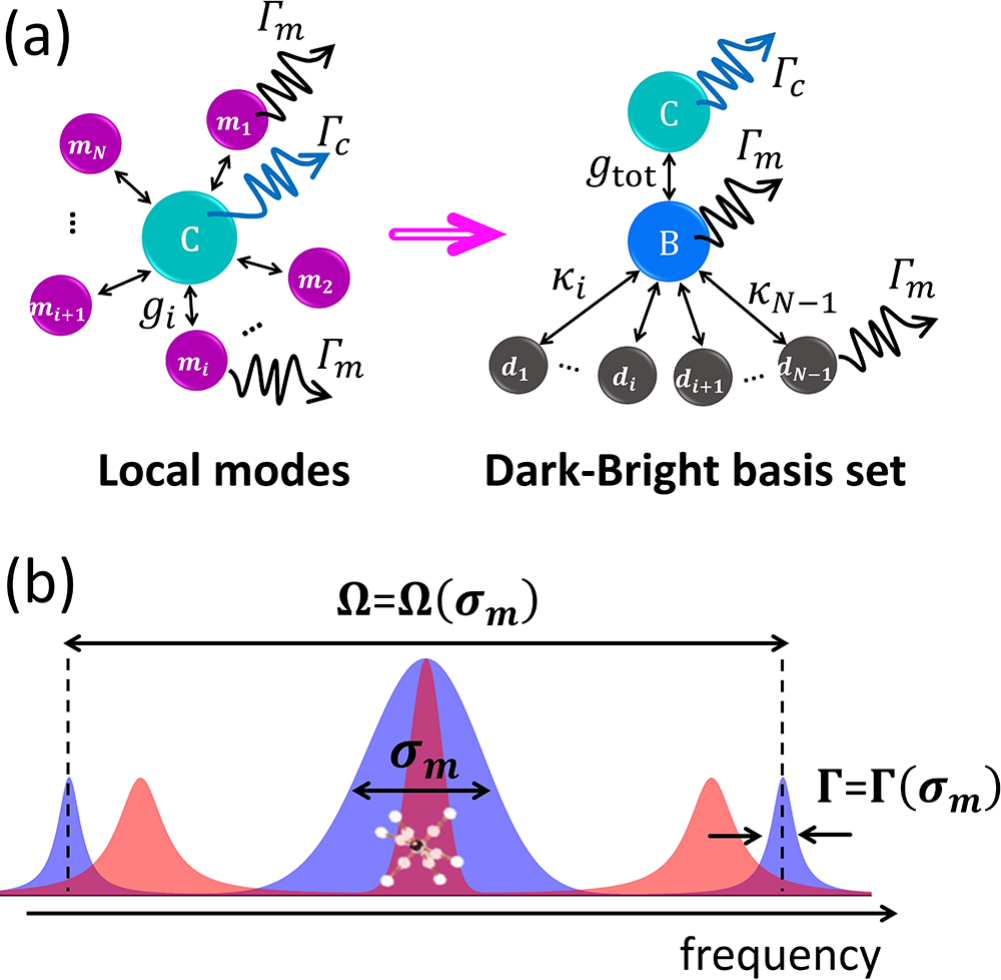
(a) Schematic illustration of the transformation of the TC-like Hamiltonian in eq 1 from the local state representation (left) to the dark–bright states representation (right). See the text for the parameter definitions. (b) Schematic illustration of changes in the spectrum of the coupled system with a moderate increase in the molecular disorder, quantified by the inhomogeneous bandwidth . The homogeneous (red) and inhomogeneous (blue) molecular ensembles are shown.

The eigenstates of the Hamiltonian (eq 1) involve the upper (UP) and the lower (LP) polariton states and the reservoir states . When , Γ_*m*_, σ_*m*_, and , the effect of  can be neglected, and the system follows the homogeneous limit.^26^ In such a regime,  are often referred to as “dark” because their transition dipoles vanish.^17,34^ Since  is negligible,  do not involve any cavity component, lack dispersion, and are localized. Furthermore, in this regime, polaritons appear to be insensitive to the molecular disorder and feature a homogeneous line shape whose bandwidth is given by . In contrast, when a homogeneous regime does not apply, the disorder can be viewed as a perturbation that facilitates admixing of the cavity mode into reservoir via the coupling constants , which lifts the reservoir’s dark character and delocalizes the quantum states. We refer to this as an inhomogeneous regime.

In the present work, we examined the role of disorder on polaritons formed by the carbonyl stretching (CO) vibrational modes of W(CO)_6_ molecules in solution, which were coupled to an open cavity. Our experimental system allows for the independent systematic control of both the molecular inhomogeneous bandwidth and its collective coupling strength to the cavity. The former is achieved via controlling solute–solvent interactions by changing the solvent polarity,^35,36^ whereas the latter is achieved via controlling the concentration of solute molecules.^37^ We demonstrate that, counterintuitively, in the inhomogeneous regime, an increase in molecular disorder leads to an increase in polariton splitting and line width modification, as schematically illustrated in Figure 1b. With the help of the theoretical analysis, we further show that both observations arise from the disorder-induced admixture of the cavity mode into the reservoir manifold. We quantify this effect with the effective coupling constant , which describes the coupling between the bright and dark states, and we demonstrate its correlation with the degree of delocalization of the reservoir states.

Experimentally, an open cavity was constructed with a high-quality antenna-lattice resonance (ALR) of the half-wavelength infrared antenna array,^38,39^ where individual dipolar antenna resonances couple to the in-plane lattice diffraction order.^40,41^ Unlike the commonly used Fabry–Perot-style optical cavities, in our experiments, the optical extinction occurs only at the ALR frequency while the nearby spectral region remains transparent, thus allowing direct spectroscopic access both to the polaritons and to the reservoir transitions.^38^ A 3 × 3 mm nearly defect-free antenna array was fabricated by means of electron-beam lithography on a CaF_2_ window.^42−44^ The gold antennas (dimensions of 0.75 × 0.2 × 0.07 μm) were arranged in a rectangular lattice with a longitudinal period (along the antenna axis) of *D*_L_ = 1.35 μm and a transverse period of *D*_T_ = 3.8 μm, as shown in Figure 2a (inset). The linear extinction spectra, collected at a normal incidence of light polarized along the antenna axis, are shown in Figure 2a for an array immersed in the index-matching (nonpolar) *n*-octane and (polar) 1-chlorobutane solvents. In both solvents, the ALR appears at the same frequency of  cm^–1^ and has a quality factor of *Q* ∼ 115. The choice of the two fully miscible solvents with similar refractive indices but with different polarities allows us to efficiently control the molecular disorder without modifying the frequency or bandwidth of the ALR.

**Figure 2:**
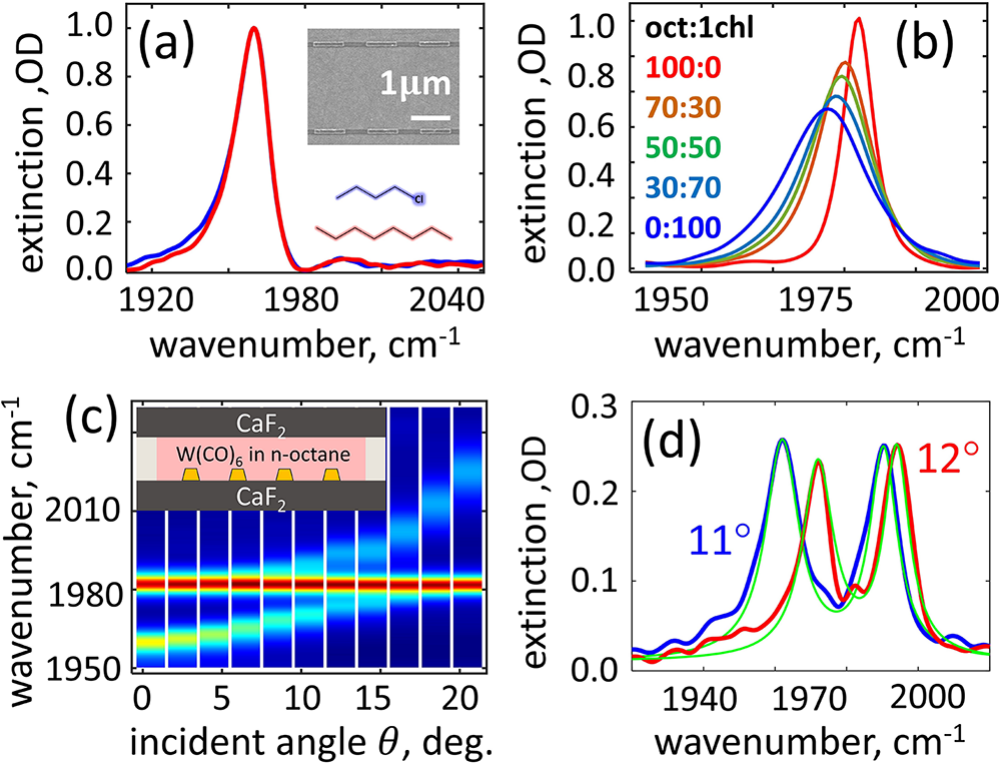
Vibrational polaritons in an open cavity. (a) The extinction spectrum of the ALR at normal incidence in index-matching *n*-octane (red) and 1-chlorobutane (blue) solvents. The inset shows the scanning electron microimage of the antenna array. (b) The carbonyl stretching vibrational mode of 20 mM W(CO)_6_ solutions in *n*-octane/1-chlorobutane mixtures. The color code is shown on the left. (c) Polariton dispersion in *n*-octane. The strongest coupling to molecular vibrations is achieved at an incident angle of 12°. (d) The background-subtracted polariton extinction in *n*-octane (red) and 1-chlorobutane (blue). In 1-chlorobutane, the strongest coupling was achieved at 11°. Green lines show fits of the polariton spectrum to a sum of two Lorentzian profiles.

The molecular inhomogeneous bandwidth was systematically varied by preparing different mixtures of *n*-octane and 1-chlorobutane. As shown in Figure 2b, when the fraction of 1-chlorobutane increases, the CO transition broadens and red-shifts from  = 1982 cm^–1^ to  = 1977 cm^–1^ (vibrational Stark effect^35,36^). The broadening reflects increases in both the homogeneous and inhomogeneous line shape components with the increasing solvent polarity. The increase in inhomogeneity indicates the presence of multiple solvation shell configurations created by 1-chlorobutane around the CO group. The spectra fit well with the Voigt line shape, which was previously shown to describe the dynamics of CO transition in W(CO)_6_ in aliphatic solvents.^45,46^ However, because fitting the linear spectrum to the Voigt profile may produce homogeneous and inhomogeneous widths with a high covariance, we determined their values for each solvent mixture using 2D IR spectroscopy, as described in details in the Supporting Information. The results are shown in Figures S1 and S2, and all the obtained parameters are summarized in Table S1. Changing the solvent composition from *n*-octane to 1-chlorobutane leads to an increase in the inhomogeneous bandwidth from  = 1.1 cm^–1^ to  = 4.4 cm^–1^, whereas the homogeneous bandwidth changes from  = 3.9 cm^–1^ to  ≈ 7 cm^–1^.

The antenna array was covered by a 3.6 μm- thick 20 mM solution of W(CO)_6_ and capped with an additional CaF_2_ window. The dispersion of the vibrational polaritons measured for different incident angles, shown in Figure 2c, features an avoided crossing between the ALR and the CO modes at around  in neat *n*-octane and that at  in neat 1-chlorobutane. To compensate for the solvent-induced shift of the CO mode frequency and to maintain the resonant conditions, the ALR transition was tuned without affecting the ALR quality by slightly changing the angle of the TM-polarized incident light^39^ with a precision goniometer, as shown in Figure S3 of the Supporting Information.

The background-subtracted spectra of the strongly coupled system are shown in Figure 2d for the two neat solvents. The background spectra were collected for each sample away from the array. Background subtraction allows for both the efficient removal of the contribution from the uncoupled W(CO)_6_ molecules^47,48^ and the detailed resolution of the polariton line shapes (see also Figure S4 of the Supporting Information). Polariton spectra were fit to two Lorentzian profiles, and the vacuum Rabi splitting  was calculated from the peaks’ central frequencies obtained for the solvent mixtures of different compositions, as plotted in Figure 3a. To keep the coupling strength  constant, the concentration was kept at 20 mM in all samples. Using Beer’s law, we verified that the extinction coefficient of W(CO)_6_ does not differ significantly in these solvents, as demonstrated in Figure S5 of the Supporting Information.

**Figure 3:**
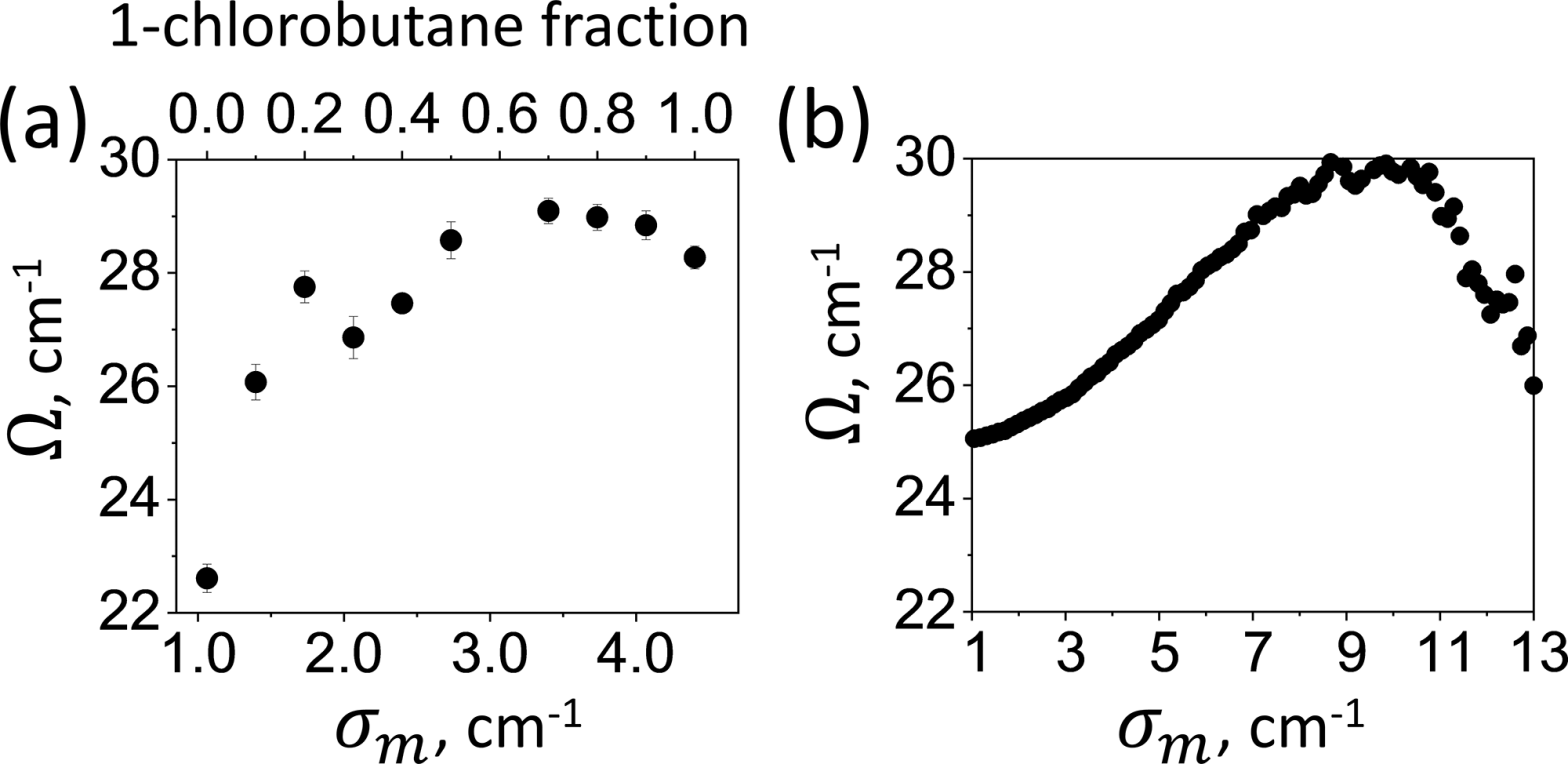
Disorder dependence of the Rabi splitting. (a) Experimental  values plotted for different  values (bottom abscissa). The corresponding solvent composition (1-chlorobutane fraction) is shown in the top abscissa. The error bars show the uncertainties in  calculated from the uncertainties in fit parameters. (b) Calculated  values obtained with eq 1 using experimentally determined line-shape parameters; *N* = 5000 and  = 26 cm^–1^.

Remarkably, changing the solvent polarity leads to increase in  by up to ∼35%. While both  and  affect the value of  (see eq 2 below),  accounts only for a small fraction of the observed . As shown in Figure 3a, the maximum splitting was obtained for  ≈ 3.5 cm^–1^, which was followed by a slight decrease. In neat solvents, the Rabi splittings were  = 22.8 cm^–1^ and  = 28.5 cm^–1^. These splitting values exceed the bandwidth of the uncoupled transitions of both the ALR ( =  = 18 cm^–1^, fwhm) and the total bandwidth of the CO mode ( = 5.1 cm^–1^ and  = 13.7 cm^–1^) by less than 10-fold, indicating the inhomogeneous regime, as discussed above. The increase in the Rabi frequency is qualitatively reproduced by the real parts of the eigenvalues of the Hamiltonian (eq 1),^49^ accounting for the experimentally determined , , and  values, as shown in Figure 3b. The numerical model predicted  values similar to the experimental ones, but the maximal splitting was obtained for  = 9 cm^–1^, which was larger than that in the experiment.

To facilitate an intuitive interpretation of the results in Figure 3, we reduced the bright–dark state representation of the Hamiltonian in eq 1 to an effective three-state model, which is schematically illustrated in Figure 4a. Here, the cavity mode is coupled to the bright state  by  as described earlier, whereas the dark states are represented by a single surrogate collective state  coupled to  with the effective rate , where the values depend on the disorder .^19^ The new Hamiltonian is
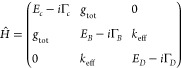


**Figure 4:**
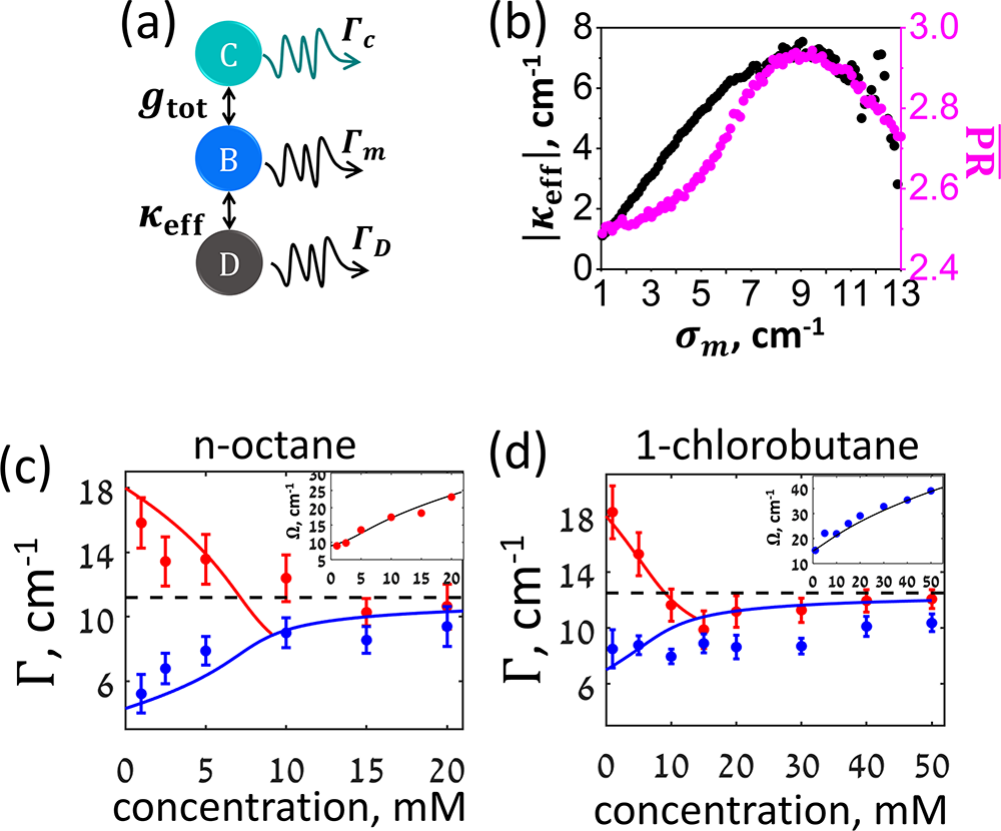
(a) A three-state model reduced from the bright–dark representation of the TC-like Hamiltonian in eq 1. (b) The dependence of  and  (the average participation ratio of the reservoir states) on the molecular disorder. (c, d) Modification of the polariton bandwidth in *n*-octane and in 1-chlorobutane, respectively. Red represents , blue represents , dots represent the experimental data, lines represent the results of a three-state model, and horizontal dashed lines represent . Significant hybridization emerges around the exceptional point, where the bandwidths of the hybrid states become comparable. Insets show the corresponding  values.

Because the collective  and  states are purely molecular, the energies of these states are  =  =  (at the resonance,  is also equal to ), the corresponding decay rates are  = , and  can be chosen to effectively represent the bandwidth of the molecular transition.

An approximate analytical solution for the complex eigenvalues, namely, , can be obtained if we further assume that . Although such an approximation is certainly not valid for analyzing the reservoir modes, we find it useful for analyzing polaritons.^19^ Under these approximations, the real part of the eigenvalues is 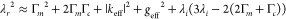
Assuming that the bandwidth of the polaritons does not differ significantly from that in the homogeneous limit, namely, , we obtain for the vacuum Rabi splitting
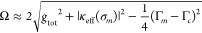
2

Substituting  and  values obtained by diagonalization of the Hamiltonian in eq 1 into eq 2 allows the coupling strength constant  to be extracted for various  values, as shown in Figure 4b. For low-to-moderate disorders,  grows, reaching the maximal value at  ≈ 9 cm^–1^, where  is the largest. For larger disorder,  decreases, leading to a decrease in .^19^

Next, we analyze the polaritons’ bandwidths. For the 20 mM solutions, bandwidths of  = 9.4 cm^–1^,  = 10.6 cm^–1^,  = 8.6 cm^–1^, and  = 11.2 cm^–1^ were obtained for neat *n*-octane and 1-chlorobutane. Interestingly, for all transitions, the bandwidths were below those expected in the homogeneous limit, where  = 11.6 cm^–1^ and  = 12.6 cm^–1^. To examine polariton line narrowing, we measured the change in the bandwidth for different  values, which were obtained with different concentrations of the W(CO)_6_ molecules. As shown in Figure 4, as the coupling strength increases ,  decreases from the bare-cavity value  = 18 cm^–1^ (for  = 0) to  = 10.6 cm^–1^ and  = 12.0 cm^–1^, which are below the expected  and  values. On the other hand,  increase from the bare-molecule values of =4 cm^–1^ and =7 cm^–1^ toward  and ; however, Γ_UP_^oct^ and Γ_UP_^1chl^ do not reach the homogeneous values within the range of the molecular concentrations used in our experiments, which were limited by the W(CO)_6_ solubility. These experimental observations were qualitatively reproduced by the imaginary part of the eigenvalues of the Hamiltonian in eq 1. The reduced three-state model also captures the experimental results, as shown in Figure 4 for  = 4.5 cm^–1^ and  = 6 cm^–1^.

Agreement between the experimental and theoretical results allowed us to use the latter to explore why there was an increase in Rabi splitting with an increase in the molecular disorder and the modification of polariton line widths with an increase in the coupling strength. To this end, we inspected the compositions of the corresponding eigenstates  +  +  and  +  where  are the Hopfield coefficients describing the admixture of the unperturbed state  into the eigenstate , as shown in Figure 5. Generally, with the increase in inhomogeneity, the admixing of the cavity mode into polaritons decreases, whereas the admixing of the corresponding molecular component increases. In our experiments, , which leads to narrowing of the polariton lines below the  value, as corroborated by the correlation between and  with  and , respectively, as shown in Figure 5. Analogously, for , the broadening of polariton lines beyond the  is expected.

**Figure 5:**
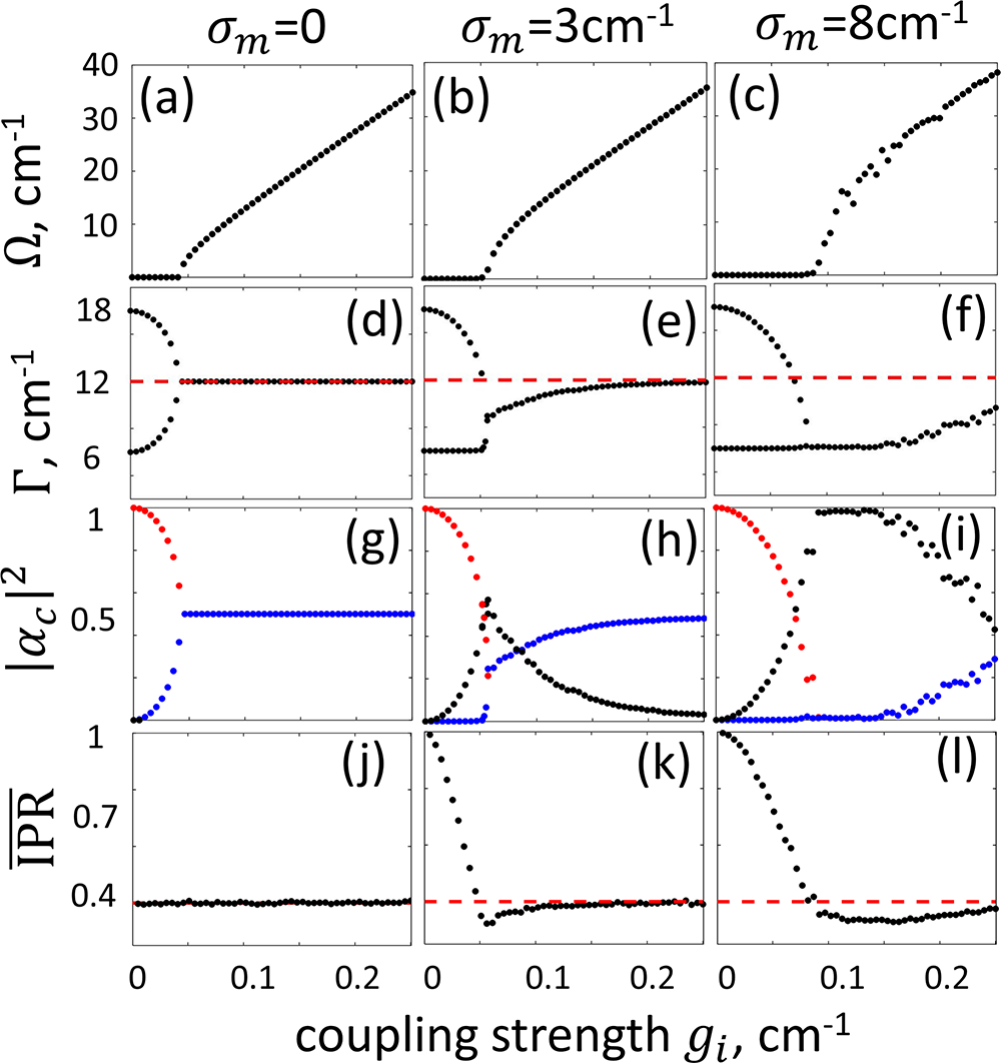
Numerical solution of the non-Hermitian TC-like Hamiltonian. (a–c) Vacuum Rabi splitting and (d–f) imaginary parts of the polariton eigenvalues obtained for different coupling strength constants , with *N* = 5000, ,  = 18 cm^–1^, and  = 6 cm^–1^. The left column represents a homogeneous molecular ensemble, the middle column represents weak disorder of the molecular ensemble, and the right column represents strong disorder of the molecular ensemble. The corresponding inhomogeneous bandwidth is indicated above each column. Red dashed lines in panels d–f show the  value. (g–i) Hopfield coefficients quantifying the cavity contribution to the eigenstates. Red, blue, and black dots show , , and , respectively. (j–l) Inverse participation ratio averaged over the reservoir states, where  = 1/. The red dashed line represents the corresponding homogeneous value  = 0.4.

While naturally  = 0 for  = 0, for  ≠ 0, an admixture of the cavity and the reservoir leads to the delocalization of the  states, where the degree of delocalization depends on the interplay between  and . The degree of delocalization is frequently quantified by the corresponding participation ratio (PR), which for the local states *j* delocalized over the eigenstate  is given by^50,51^
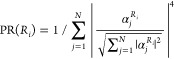


The average of PR over all the reservoir states in the homogeneous case is  = 2.5.^20,25^ For the coupling strength equivalent to that of the 20 nm W(CO)_6_ solutions, with an increase in disorder,  closely follows the trend of the change in  with , as shown in Figure 4b. Here,  increases, reaching a maximal value of ca. 3 for  ≈ 9 cm^–1^ (maximal PR = 55, std = 2.3), then decreases toward the localized states when  is insufficient to overcome the disorder.

PR depends on the interplay between  and . In the case of a weak disorder, when  increases,  briefly increases, reaching the value of  ≈ 3 near the exceptional point where polariton splitting emerges,^52^ and then approaches  (see Figure 5). As expected, the coupling strength required to surpass the exceptional point is larger in the case of stronger disorder;^21,53^ however, the region of  ≈ 3 is extended over a larger range of  values, compared with the case of weak disorder. Interestingly, strongly disordered ensembles involve fewer reservoir states delocalized over a large number of molecules compared to weakly disordered ensembles. This can be seen from the PR statistics in both cases (*N* = 40 000), which show that for  = 3 the standard deviation of the PR values distribution is ∼8 for  = 3 cm^–1^ but only ∼3 for  = 8 cm^–1^. The maximally delocalized states have PR values of 370 and 165.

Our calculations suggest that delocalization of reservoir modes is closely correlated with the admixture of the cavity mode (see Figure 5). For both cases of both weak and strong disorder, high  values were observed when the Hopfield coefficients  were high. Since in our experiments , we also consistently found that , which supports the hypothesis that the delocalization of reservoir modes is enhanced due to the cavity admixture.

In conclusion, we demonstrated that disorder within the molecular ensemble, which strongly interacts with the optical cavity, affects the polariton spectrum, leading to a larger Rabi splitting and a modified polariton bandwidth. We also showed that these phenomena are correlated with the delocalization of the reservoir states, which is enhanced by the admixture of the photonic mode into reservoir. Our experimental results, supported by theoretical models, indicate that in the regime of a moderate disorder, coupling to the cavity can allow the well-known effect of localization to be overcome. Thus, our results provide new insights into the strong interaction between matter and vacuum fields, which can be harnessed and utilized in future quantum technologies.

## Notes

The data that support the findings of this study are available from the corresponding author upon reasonable request.

## Supporting Information Available

Linear and third-order nonlinear spectra of W(CO)_6_ molecules, concentration dependence of linear signals, angle-tuning of the ALR resonances, raw and background-subtracted absorption spectra of the polariton states, and results of the Voigt line shape analysis of molecular transitions for all solvent mixtures used in the experiments (PDF)
