# Supplementary material for: Vibrational Polaritons in Disordered Molecular Ensembles
Source: J Phys Chem Lett. 2022 Aug 31;13(35):8369–75. doi: 10.1021/acs.jpclett.2c02341 (PMC9465717; doi:10.1021/acs.jpclett.2c02341)
Supplement: Supplementary file 1 — jz2c02341_si_001.pdf [file jz2c02341_si_001.pdf]

Vibrational Polaritons in Disordered Molecular Ensembles

Bar Cohn,[a,b], Shmuel Sufrin,[b,c], Arghyadeep Basu [a,b] and Lev Chuntonov[a,b,*]

[a]Schulich Faculty of Chemistry, [b]Solid State Institute, [c]Faculty of Mechanical Engineering,

Technion – Israel Institute of Technology, Haifa 3200003, Israel

*) Corresponding author; email: chunt@technion.ac.il

Supporting Information

Measurements of $W(CO)_6$ line shape components

Despite the linear line shapes of the CO stretching modes of $W(CO)_6$ fit well to Voigt profiles, the homogeneous and inhomogeneous widths obtained from these fits may have a large covariance, making their values less reliable. Thus, in order to obtain these values with higher confidence, we resorted to the third-order nonlinear time-resolved two-dimensional infrared spectroscopy (2DIR), where we extracted the homogeneous and inhomogeneous components of the spectral profiles from measurements of the frequency fluctuation correlation function (FFCF) using procedures of refs.[1,2]

Experimentally, three 70fs mid-infrared laser pulses enumerated by their wave vectors $\vec{k}_{1-3}$ were generated with the laser system (Solstice, Spectra Physics; TOPAS and DFG, Light Conversion) and focused at the sample in a box configuration. The signals emitted into the phase-matched directions $\vec{k}_s = \mp\vec{k}_1 \pm \vec{k}_2 + \vec{k}_3$ were heterodyned by the fourth pulse serving as local oscillator and measured with the MCT array detector (64 elements, Infrared Associates) for various time intervals between the $\vec{k}_1$, $\vec{k}_2$, and $\vec{k}_2$ pulses to obtain purely-absorptive 2DIR spectra.[3] Representative 2DIR spectra collected at the early and late waiting times for the neat octane and 1-chlorobutane solvents are shown in Figure S1. The waiting time dependence of the central line slope (CLS) of the fundamental transition shown in panels (c) and (d) of the figure was used as a measure of the normalized FFCF.[1]

We assumed that FFCF has the functional form of $C(t) = \frac{\delta(t)}{T_2} + \Delta_0^2 + \Delta_1^2 e^{-t/\tau_c}$, where T_2^{-1} is the homogeneous dephasing rate, Δ_0 is the standard deviation of the transition frequencies distribution associated with static inhomogeneity, Δ_1 is the amplitude of the frequency fluctuations, and τ_c is the associated correlation time. The latter term describes the process known as spectral diffusion, and is interpreted as a dynamic inhomogeneity. With the FFCF specified above, the line shape of the linear absorption is given by

$$S^{(1)}(\omega) \propto \Re\left\{\int_0^\infty \mu_{01}^2 e^{-t/T_2 - \frac{1}{2}\Delta_0^2 t^2 - \Delta_1^2 \tau_c (e^{-t/\tau_c} - t/\tau_c - 1)} e^{i(\omega - \omega_{01})t} dt\right\}, \quad \text{(S1)}$$

where μ_{01} is the transition dipole moment of the fundamental excitation with radial frequency ω_{01}, and the bandwidth of the homogeneous component of the absorption line shape is $\Gamma = \frac{1}{\pi T_2}$.

In a typical analysis,[1,2] the CLS is assumed to represent the normalized inhomogeneous part of the FFCF, $\tilde{C}(t) = \tilde{\Delta}_0^2 + \tilde{\Delta}_1^2 e^{-t/\tau_c}$, where $\tilde{\Delta}_0^2$ and $\tilde{\Delta}_1^2$ are the coefficients of the associated contributions to $\tilde{C}(t)$. When $\tilde{C}(t)$ is obtained from the CLS experimental data, the homogeneous bandwidth Γ can be either estimated from $\Gamma/\text{FWHM} \approx 1 - \left(\tilde{\Delta}_0^2 + \tilde{\Delta}_1^2\right)$, where FWHM is the full width at half maximum of the total spectral profile, or obtained from fitting the linear spectrum to equation S1.[1] Finally, the inhomogeneous part of the line shape is estimated from the approximate expression for the Voigt profile,[4] $\text{FWHM} \approx \frac{\Gamma}{2} + \sqrt{\frac{\Gamma^2}{4} + (8\ln(2)\,\sigma_m)^2}$, where σ_m is the effective inhomogeneous bandwidth. Results for all the solvent mixtures are summarized in Table S1; the Γ and σ_m values are plotted in Figure S5.

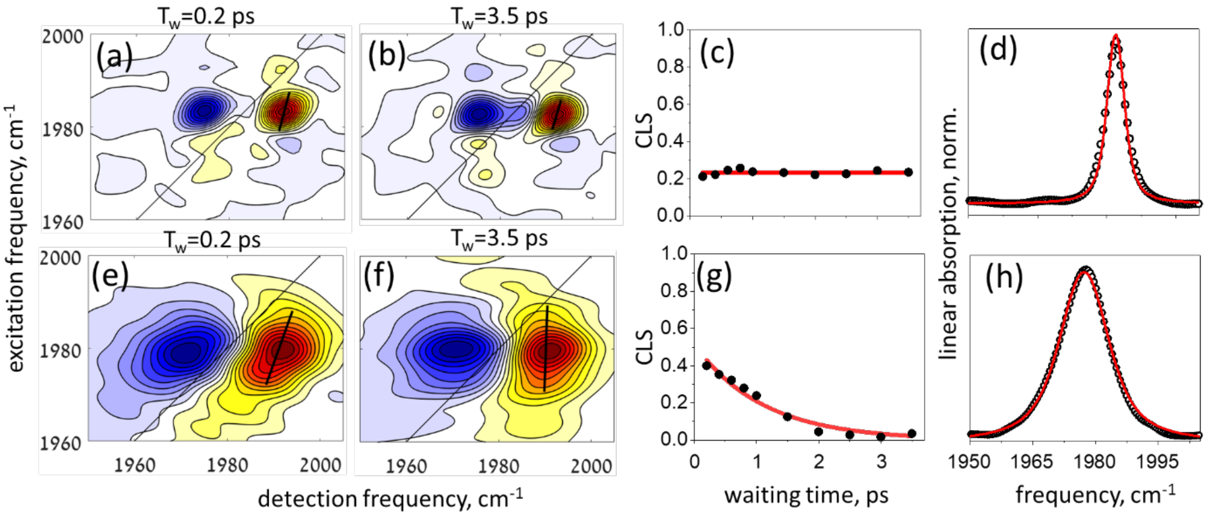

Figure S1. Spectroscopy of $W(CO)_6$ in n-octane (a-d) and 1-chlorobutane (e-h). 2DIR spectra at early (a, e) and late (b, f) waiting times. The CLS of the fundamental transition is shown with a solid line; waiting times are indicated above each spectrum. (c, g) The waiting time dependence of the CLS. (d), (h) Linear spectra (circles) and fits (red lines) to the line profile of equation S1.

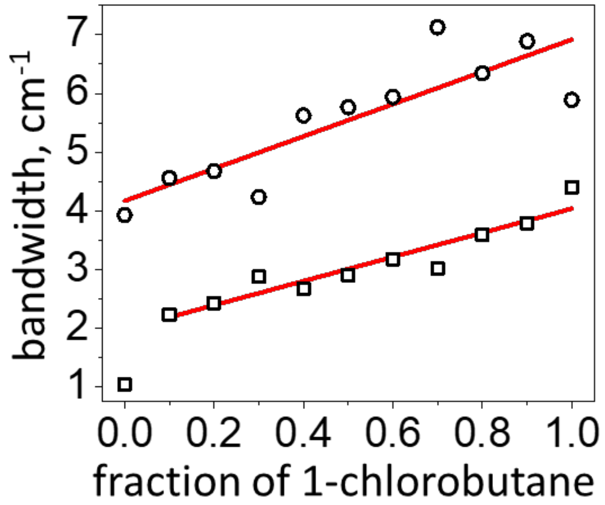

Figure S2. Line shape components of the CO transition. Circles - Γ, squares - σ_m. Red lines show linear trends.

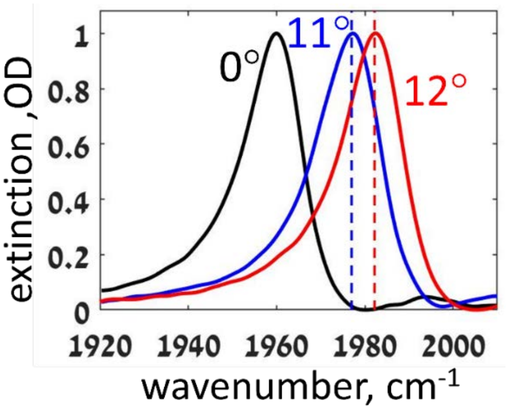

Figure S3. Incident-angle tuning of ALR. Black line - extinction spectrum of the ALR at normal incidence. The CO transition frequencies are shown in dashed vertical lines for n-octane (red) and 1-chlorobutane (blue). Resonant conditions are obtained by changing the angle of incidence to 12° in n-octane and 11° in 1-chlorobutane.

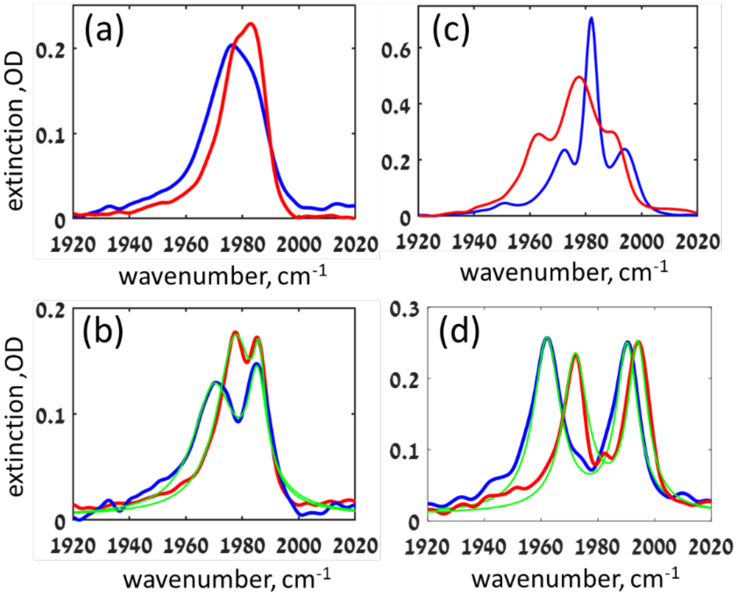

Figure S4. Extinction spectrum of vibrational polaritons. (a), (c) – raw spectra; (b), (d) - spectra after the background subtraction. Red lines - n-octane, blue lines - 1-chlorobutane solutions. The $W(CO)_6$ concentration is 1 mM in (a) and (b), and 20 mM in (c) and (d). Green lines - fits to a sum of two Lorentzian profiles. Note, that for 1 mM samples, the hybridization is not complete, with the corresponding Hopfield coefficients $|\alpha_c^{\mathrm{LP}}|^2$=0.95 and $|\alpha_c^{\mathrm{UP}}|^2$=0.025.

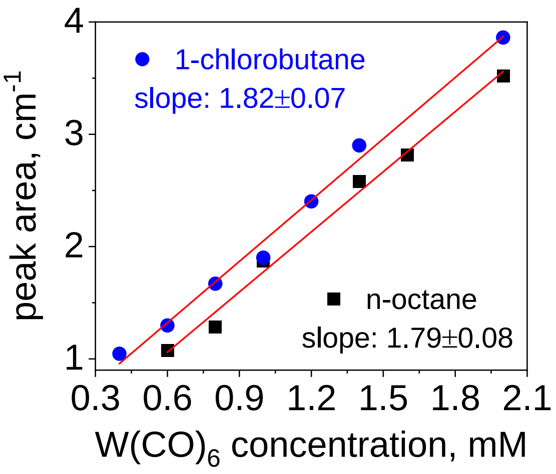

Figure S5. Concentration dependence of the CO stretching mode absorption signal area. The slope of the linear regression line is converted into the integrated absorption coefficient (IAC=$\int \varepsilon(\omega)d\omega$) using Beer's law, $A = \int \varepsilon(\omega)d\omega\, CL$. Here, A is the total absorption (peak area), C is the molecular concentration, and L=30 μm is the path length, such that IAC=$6.1\times10^5\pm2\times10^4 M^{-1}cm^{-2}$ in 1-chlorobutane and $6.0\times10^5\pm2\times10^4$ $M^{-1}cm^{-2}$ in n-octane.

Table S1. Line shape analysis of molecular transitions

1-cholrobutane fraction, %	ω_{CO}, cm^{-1}	FWHM, cm^{-1}	Γ_m, cm^{-1}	$\tilde{\Delta}_0^2$, ps^{-2}	$\tilde{\Delta}_1^2$, ps^{-2}	τ_c, ps	σ_m, cm^{-1}
0	1981.9	5.1	3.9	0.23	0	-	1.1
10	1981.1	8.0	4.6	0.19	0.24	1.7	2.2
20	1980.1	8.5	4.7	0.14	0.40	1.3	2.4
30	1980.0	9.2	5.2	0.15	0.29	0.8	2.9
40	1979.4	9.7	5.6	0.08	0.35	0.8	2.7
50	1979.8	10.3	5.8	0.05	0.39	0.7	2.9
60	1978.9	11.0	5.8	0	0.46	0.9	3.2
70	1978.8	11.5	7.1	0	0.38	0.9	3.0
80	1978.5	12.2	6.5	0	0.50	1.2	3.6
90	1978.5	13.0	6.9	0	0.48	1.5	3.8
100	1977.3	13.7	5.9	0	0.57	1.0	4.4

References

(1) Kwak, K.; Park, S.; Finkelstein, I. J.; Fayer, M. D. Frequency-Frequency Correlation Functions and Apodization in Two-Dimensional Infrared Vibrational Echo Spectroscopy: A New Approach. *J. Chem. Phys.* **2007,** *127*, 124503.

(2) King, J. T.; Baiz, C. R.; Kubarych, K. J. Solvent-Dependent Spectral Diffusion in a Hydrogen Bonded “Vibrational Aggregate”. *J. Phys. Chem. A* **2010,** *114*, 10590-10604.

(3) Khalil, M.; Demirdöven, N.; Tokmakoff, A. Obtaining Absorptive Line Shapes in Two-Dimensional Infrared Vibrational Correlation Spectra. *Phys. Rev. Lett.* **2003,** *90*, 047401.

(4) Whiting, E. E. An Empirical Approximation to the Voigt Profile. *Journal of Quantitative Spectroscopy and Radiative Transfer* **1968,** *8*, 1379-1384.
